# Influence of Electrolyte Conductivity on Energy Consumption and the Oxide Films Performance During Anodic Oxidation of Aluminum Electrolytic Capacitors

**DOI:** 10.3390/ma19184019

**Published:** 2026-09-21

**Authors:** Yubin Cai, Pengfei Liu, Hang Dong, Yuan Guo, Tao Hu, Yi Yan

**Affiliations:** 1 School of Mechanical Engineering, Xinjiang University, Urumqi 830049, China; 2 School of Materials Science and Engineering, Xinjiang University, Urumgi 830049, China; 3 Center for Post-Doctoral Studies of Mechanical Engineering, Xinjiang University, Urumgi 830049, China

**Keywords:** anode foil, multi-step formation, conductivity, energy-saving

## Abstract

Anode foil is a key component of aluminum electrolytic capacitors, and its performance largely depends on the anodic oxidation, or formation, process. During formation, electrical current converts the aluminum surface into a thin insulating oxide film that determines the capacitor’s voltage resistance and reliability. Electrolyte conductivity controls how easily ions move through the solution: excessively low conductivity increases resistive energy losses, whereas excessively high conductivity can cause electrical discharges that damage the oxide film. This study investigated the effects of low (800 μS·cm^−1^), medium (1500 μS·cm^−1^), and high (2700 μS·cm^−1^) conductivities on energy consumption and oxide-film quality during formation at 520 V. The corresponding energy consumptions were 761.635, 707.381, and 754.238 kJ, respectively. Medium conductivity achieved the lowest energy consumption, representing reductions of 7.1% and 6.2% compared with low and high conductivities, respectively. However, the oxide film formed at medium conductivity showed lower crystallinity, revealing a trade-off between energy efficiency and film quality. Based on these results, a four-stage process was developed by applying high conductivity at ≤300 V, medium conductivity at 300–400 V, and low conductivity at 400–520 V. Compared with the conventional single-stage process, the optimized process reduced energy consumption by 8.4% and increased the voltage rise rate by approximately 6%. This voltage-dependent conductivity strategy provides a practical approach to producing high-quality anode foil with lower energy consumption.

## 1. Introduction

In the context of increasing global attention to climate change and environmental sustainability, energy-efficient manufacturing has emerged as a critical area that seeks to harmonize industrial development with ecological preservation [1,2,3,4,5,6]. Consequently, the reduction in energy consumption and carbon emissions in production processes has become a paramount challenge for the global manufacturing sector.

Capacitors are essential electronic components within circuit systems [7,8], with the anode foil serving as a crucial element in their construction [9,10,11,12]. In 2023, the global demand for anode foil reached 22.44 billion square meters. The electrical energy required for the formation of each square meter of anode foil can vary significantly, ranging from several tens to several hundreds of kilowatt-hours (kW·h) [13,14,15], depending on the specific formation technique utilized [16]. The formation process is a critically important phase, as it can directly influence the quality of the anode foil [17]. Alwitt et al. [18] found that hydrating aluminum foil with hot water prior to anodic oxidation not only enhances the thickness and crystallinity of the anodic oxide layer, resulting in higher quality oxide films, but also increases energy efficiency during the formation process by a factor of two. This approach conserves a significant amount of electrical energy and reduces overall energy expenditure during anodic oxidation. Li et al. [19] demonstrated that the use of symmetric pulsed current, as opposed to conventional direct current power supplies, during the anodic oxidation of aluminum foil for electrolytic capacitors leads to the formation of an oxide film with greater density, improved crystallinity, superior dielectric properties, and increased specific capacitance. Consequently, this enhances the performance of the fabricated oxide film while significantly reducing energy expenditure, achieving an overall reduction in energy consumption of 25.5% compared to traditional direct current anodic oxidation methods. Ban et al. [20] revealed that an oxide film produced from a solution enriched with boric acid and citric acid exhibits superior crystallinity compared to that formed in a conventional boric acid solution; however, the outer layer of the oxide film contains a higher incidence of defects, which compromises its withstand voltage capability. Katoh et al. [21] observed that the components of the electrolytic solution have a substantial impact on both energy efficiency and the characteristics of the oxide film during the anodic oxidation process. Specifically, anodic oxidation conducted in an ammonium adipate electrolyte solution demonstrates exceptionally high energy utilization efficiency, with a current efficiency reaching 99.4%, resulting in an oxide film characterized by minimal leakage current; however, its resistance to degradation induced by hydration is relatively inadequate. Collectively, the existing literature has demonstrated that formation parameters—such as hydration pretreatment, current waveform, and electrolyte composition—substantially influence both energy expenditure and the quality of the anodic oxide film. However, most of these studies have been conducted under either fixed or narrowly varied conditions, and systematic investigations into the role of electrolyte conductivity, particularly at high formation voltages, remain surprisingly limited. Moreover, although stepwise or multistage anodizing strategies have been sporadically reported, no prior work has quantitatively examined how deliberately varying the electrolyte conductivity across different voltage stages can jointly affect energy consumption and film structural integrity.

The present study systematically investigates the evolution of formation energy consumption and oxide film characteristics at a target voltage of 520 V under three distinct conductivity levels (800, 1500, and 2700 μS·cm^−1^). On this basis, we propose a novel four-step formation protocol featuring a conductivity-switching strategy—employing a high-conductivity electrolyte during the low-voltage phase to reduce ohmic losses, and a low-conductivity electrolyte during the high-voltage phase to suppress defect formation and maintain film crystallinity. This tailored approach not only achieves a marked reduction in total energy consumption compared with conventional constant-conductivity processes, but also yields an oxide film with superior dielectric strength and structural uniformity.

## 2. Materials and Methods

### 2.1. Preparation Process of Anode Foil

Before etching, the metal sheets were cleaned with deionized water and dried. A programmable DC power supply (SOYI-1000-05, Shanghai Soyi Electronics Co., Ltd., Shanghai, China) was employed to treat etched aluminum foils (supplied by Xinjiang Joinworld Co., Ltd., Urumqi, China) that had been uniformly pretreated by hydration in deionized water at 100 °C for 10 min. The anodic oxidation (formation) was performed in a capacitor-grade boric acid solution (Shenzhen Capchem Technology Co., Ltd., Shenzhen, China) at a constant temperature of 80 °C and a formation current density of 50 mA·cm^−2^, with a total formation time of 3000 s. The electrolyte conductivity was adjusted to three distinct levels—low (800 μS·cm^−1^), medium (1500 μS·cm^−1^), and high (2700 μS·cm^−1^)—using ammonium pentaborate as the additive, and all formations were carried out to a final voltage of 520 V.

The complete process sequence for the single-stage formation was as follows: formation (50 min, 520 V) → thermal treatment (2 min, 500 °C) → additional formation (10 min, 520 V) → phosphoric acid treatment (8 min, 60 °C) → additional formation (10 min, 520 V) → drying → anode foil. Specifically, the one-step formation at varying conductivities consisted of anodizing the foil to 520 V in the boric-acid-based electrolyte and holding at this voltage for the full 3000 s. The detailed parameters for the primary formation under the three conductivity conditions are summarized in Table 1.

### 2.2. Collection of Electrical Parameters and Electrical Performance Testing

The built-in data acquisition system of the direct current power supply (SOYI-1000-05, Shanghai Soyi Electronics Co., Ltd., Shanghai, China) is employed to record current and voltage data throughout the formation process, eliminating the need for external voltmeters or ammeters. The energy values presented in this study are calculated based on the product of current and voltage (P = UI). The number of significant digits in the energy values reflects the precision of the calculations based on the measured current and voltage values. The TV tester (Shenzhen Juyinghui Technology, model TS2612, Shenzhen, China) is utilized to gather electrical performance data, including voltage rise time, withstand voltage, hydration voltage rise time, and leakage current of the formed foil samples.

### 2.3. Characterization

The surface morphology of the oxide films formed on the etched aluminum foils was characterized using a field-emission scanning electron microscope (FE-SEM, JSM-7610, NEC, JEOL Ltd., Akishima, Japan). Prior to SEM observation, all samples were sputter-coated with a thin layer of gold (or platinum) using an ion sputtering apparatus to enhance electrical conductivity and prevent charging effects during imaging.

The characterization of the specimens’ microstructure was conducted using a field-emission scanning electron microscope (Hitachi Limited, SU8010, Tokyo, Japan). For the crystallographic analysis of the specimens, a copper-target X-ray diffractometer (Bruker, Karlsruhe, Germany, D8advance) was employed. The operational voltage for the analysis was set at 40 kV, with a current of 40 mA. The scanning range for 2-Theta was established from 20° to 80°, with increments of 0.02° and a scanning rate of 10° per minute.

Electrochemical impedance spectroscopy (EIS) measurements were performed to determine the cell impedance under three electrolyte conductivity conditions. The EIS experiments were conducted using a CHI660E electrochemical workstation (Shanghai Chenhua Instrument Co., Ltd., Shanghai, China). The hydrated foils (pre-treated in hot water at 80 °C for 10 min) were used as the working electrode in a three-electrode system, with a platinum sheet as the counter electrode and a saturated calomel electrode (SCE) as the reference electrode. The measurements were conducted at 80 ± 1 °C in boric acid formation solutions with conductivities of 800, 1500, and 2700 μS·cm^−1^, consistent with the formation conditions. EIS spectra were recorded at the open-circuit potential with an AC amplitude of 10 mV over a frequency range from 100 kHz to 10 mHz, with 10 points per decade. The solution resistance (Rq) was determined from the high-frequency intercept of the Nyquist plot with the real axis. All measurements were repeated three times for each conductivity condition to ensure reproducibility.

The current study investigates the impact of electrolyte conductivity on energy expenditure and the properties of oxide films during the anode foil formation process. The objective is to optimize this process in order to reduce energy consumption and enhance the quality of the oxide films. In the one-step formation process across three distinct conductivity scenarios, an increase in conductivity results in a reduced rate of voltage increase. This phenomenon can be attributed to the diminished growth rate of the oxide film in solutions with higher conductivity. Upon reaching a voltage of 520 V and entering a constant-voltage phase, the voltage stabilizes, while the current begins to decline rapidly. During the initial stages of the constant-voltage phase, the rate of decrease in the formation current is notably swift. However, as time progresses and the anode foil formation advances into the later stages of the constant-voltage phase, the rate of current decrease becomes progressively slower. Furthermore, this rate of current decrease is directly proportional to the level of conductivity.

## 3. Results and Discussion

### 3.1. Investigation of the Influence of Conductivity on Formation Energy Consumption

The implementation of one-step formation on the foil was conducted across a range of conductivities over an area of 30 cm^2^, utilizing a current intensity of 1.5 A. The electrolytic solution was formulated by adding 50 g/L of boric acid to deionized water, while the conductivity of the solution was modulated using ammonium pentaborate. With the electrolyte conductivities established at 800, 1500, and 2700 μS·cm^−1^, the foil was subjected to a potentiation of 520 V, followed by an additional strengthening process. This study aimed to investigate the influence of conductivity on the formation procedure and operational principles, with the objective of refining the composition of the electrolyte for multi-stage formation processes.

**Figure 1:**
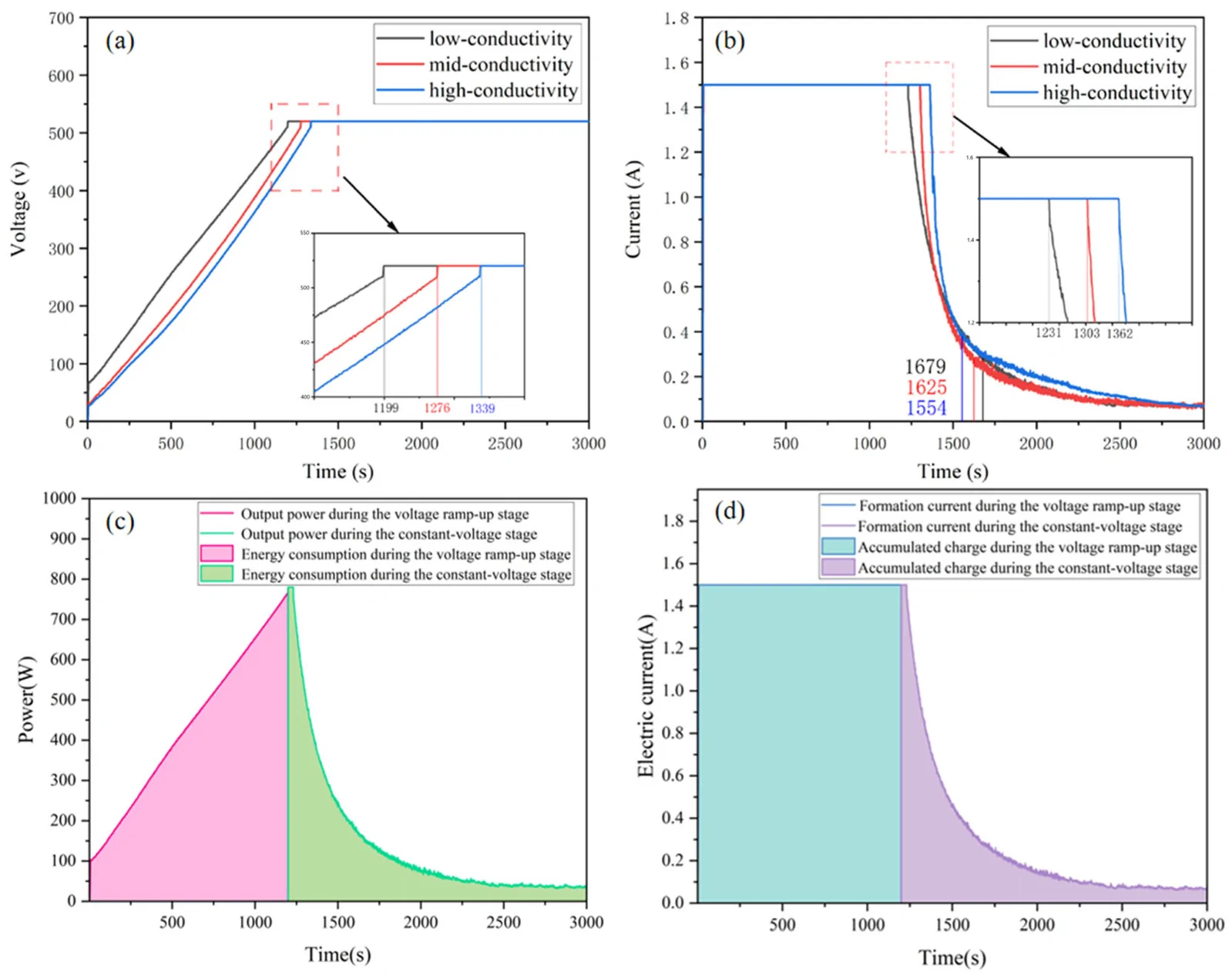
Voltage–time (**a**) and current–time (**b**) curves for formation at 800 (low), 1500 (medium), and 2700 (high) μS·cm^−1^; panels (**c**,**d**) present the energy and charge profiles during the voltage ramp-up and constant-voltage stages, respectively, under low-conductivity conditions.

This study involves the surveillance and documentation of voltage (V) and current (I) parameters throughout the formation process, utilizing the formula P (power) = UI to determine the energy expenditure during this process. The current–time and voltage–time profiles of the formation are depicted in Figure 1. The graphical representation indicates that the formation process can be broadly divided into two distinct stages: the voltage ramp-up stage, during which the current remains steady, and the voltage stabilization stage, characterized by a decreasing current. The durations required for the electrode foil to reach the nominal voltage during the voltage ramp-up phase were observed to be 1199 s for low conductivity, 1276 s for medium conductivity, and 1339 s for high conductivity. During the constant voltage phase, the time intervals necessary for the current to decrease were recorded as 1679 s for low conductivity, 1625 s for medium conductivity, and 1554 s for high conductivity (the total formation time is 3000 s). These values were calculated by subtracting the time at which the current began to decline from the total formation time. Specifically, the times at which the current began to decline were 1321 s for low conductivity, 1375 s for medium conductivity, and 1446 s for high conductivity, as indicated in Figure 1b. In the voltage ramp-up phase, the voltage exhibited a predominantly linear increase. The rates of voltage increase for the electrode foil across the three distinct conductivities were calculated to be 0.43 V/s for low conductivity, 0.40 V/s for medium conductivity, and 0.38 V/s for high conductivity. The increase in conductivity results in a gradual reduction in the rate at which the electrode foil increases in voltage. At the initial stage of anodic oxidation, high electrolyte conductivity promotes rapid ion transport and accelerates oxide film growth. However, as the applied voltage increases beyond a critical threshold, excessively high conductivity induces soft sparking and dielectric breakdown, which disrupts the film growth kinetics and effectively reduces the net oxide growth rate. Therefore, high conductivity is beneficial for accelerating oxide growth at low voltages (≤300 V) but detrimental at high voltages (≥400 V). This voltage-dependent effect forms the basis for our conductivity-switching strategy. This phenomenon is attributed to the repeated breakdown and subsequent repair of the oxide film that occurs with increasing voltage in the high-conductivity electrolyte, which consequently reduces the oxide film growth rate. During the constant voltage phase, once the voltage stabilizes at 520 V, the current begins to decline rapidly. In the initial phase of this stage, the rate of decrease in the formation current is notably high; however, as the formation process progresses, the rate of current reduction diminishes. This decrease in current is directly proportional to the level of conductivity present in the solution.

**Figure 2:**
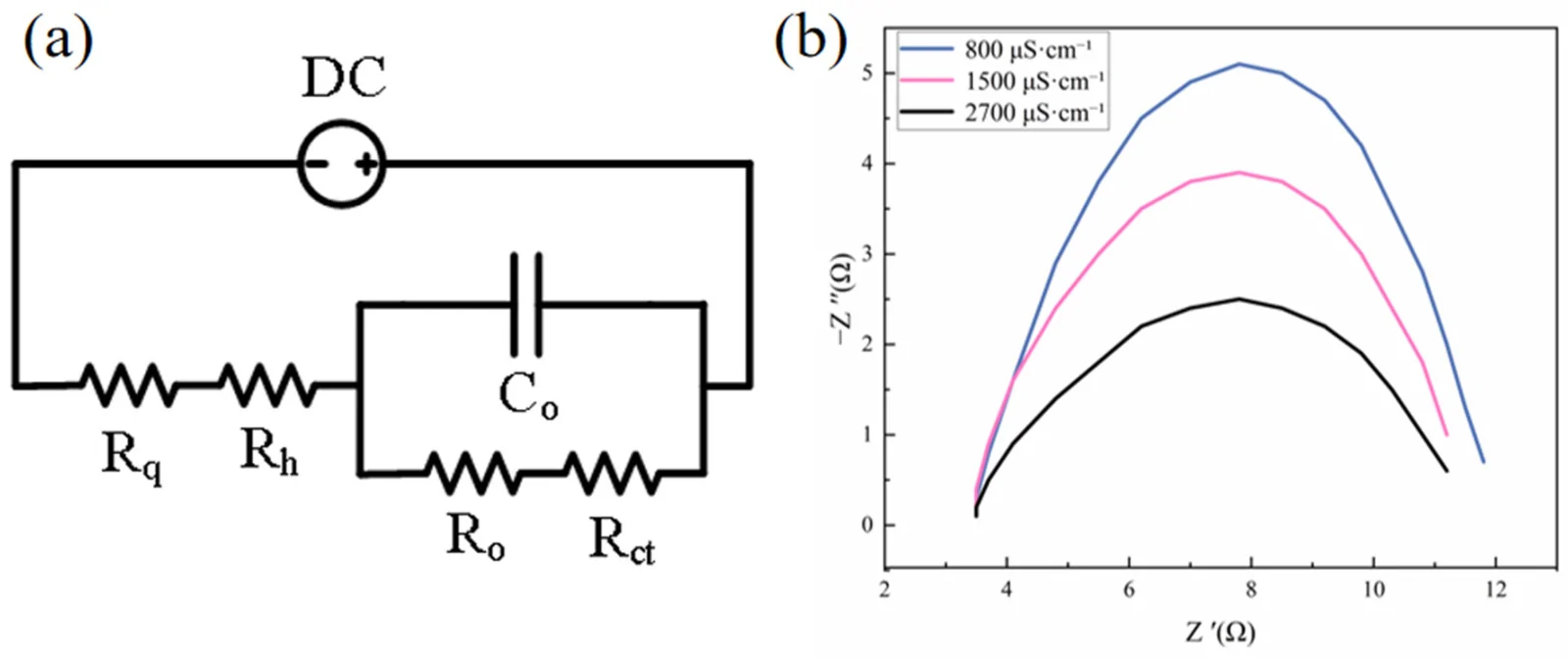
(**a**) Nyquist plots of hydrated anode foils in electrolytes with different conductivities at 80 °C. The high-frequency intercept with the real axis (Z′) represents the solution resistance (Rs). (**b**) Equivalent circuit model used for EIS data fitting.

Aluminum oxide possesses a highly compact crystalline lattice and a chemical composition of remarkable purity. As a result, it demonstrates an exceptionally high electrical resistivity [22]. During the formation process, the flow of electric current through aluminum oxide generates a substantial amount of Joule heat [23]. The electrical energy utilized in the fabrication of the oxide film is primarily directed towards the formation of the oxide film itself, as well as accounting for resistive losses that occur during the formation process. As depicted in Figure 2, an equivalent circuit model has been developed to represent the oxide film characterized by low conductivity, with the aim of elucidating the mechanisms of energy expenditure within this low conductivity formation regime. In this model, Rq represents the resistance of the solution, Rh indicates the resistance of the hydration layer, Co denotes the capacitance of the oxide film, Ro signifies the resistance of the oxide film, and Rct represents the resistance to charge transfer.

Figure 1 shows that the initial formation voltages were 69, 34, and 26 V for electrolyte conductivities of 800, 1500, and 2700 μS·cm^−1^, respectively. Under galvanostatic conditions, the measured cell voltage contains an ohmic component determined by the applied current and the electrolyte resistance. EIS measurements yielded solution resistances (Rs) of 8.6, 4.2, and 2.4 Ω for the low-, medium-, and high-conductivity electrolytes, respectively. At the applied current of 1.5 A, the corresponding (iRs) drops were 12.9, 6.3, and 3.6 V. Therefore, the higher solution resistance of the low-conductivity electrolyte makes a significant contribution to its elevated initial voltage. However, the 9.3 V difference in the (iRs) drop between the low- and high-conductivity conditions accounts for only approximately 22% of the observed 43 V difference in their initial voltages. The remaining voltage difference may include contributions from electrode polarization and the resistive response of the pre-existing hydrated layer and the developing oxide film. Because these contributions cannot be uniquely separated by the present measurements, the higher initial voltage is not interpreted as direct evidence of accelerated hydrous-oxide transformation. In addition, the larger solution resistance increases the ohmic power loss (I^2^*Rs) during the constant-current voltage-ramp stage, thereby contributing to the higher formation energy consumption at low electrolyte conductivity [24]. An increased initial voltage facilitates the proliferation of the oxide film [25], as indicated by the model through the observed increases in Rq, Rh, Co, and Ro. This enhancement results in elevated resistance during the voltage increase phase [26], consequently leading to greater energy expenditure throughout the voltage ramp-up process compared with that during the constant-voltage stage, and also a larger amount of charge transfer. In contrast to formations characterized by medium and high conductivities, a larger proportion of energy is expended to overcome the resistance of the oxide film in low-conductivity formations, and this energy is ultimately converted into heat and dissipated.

**Figure 3:**
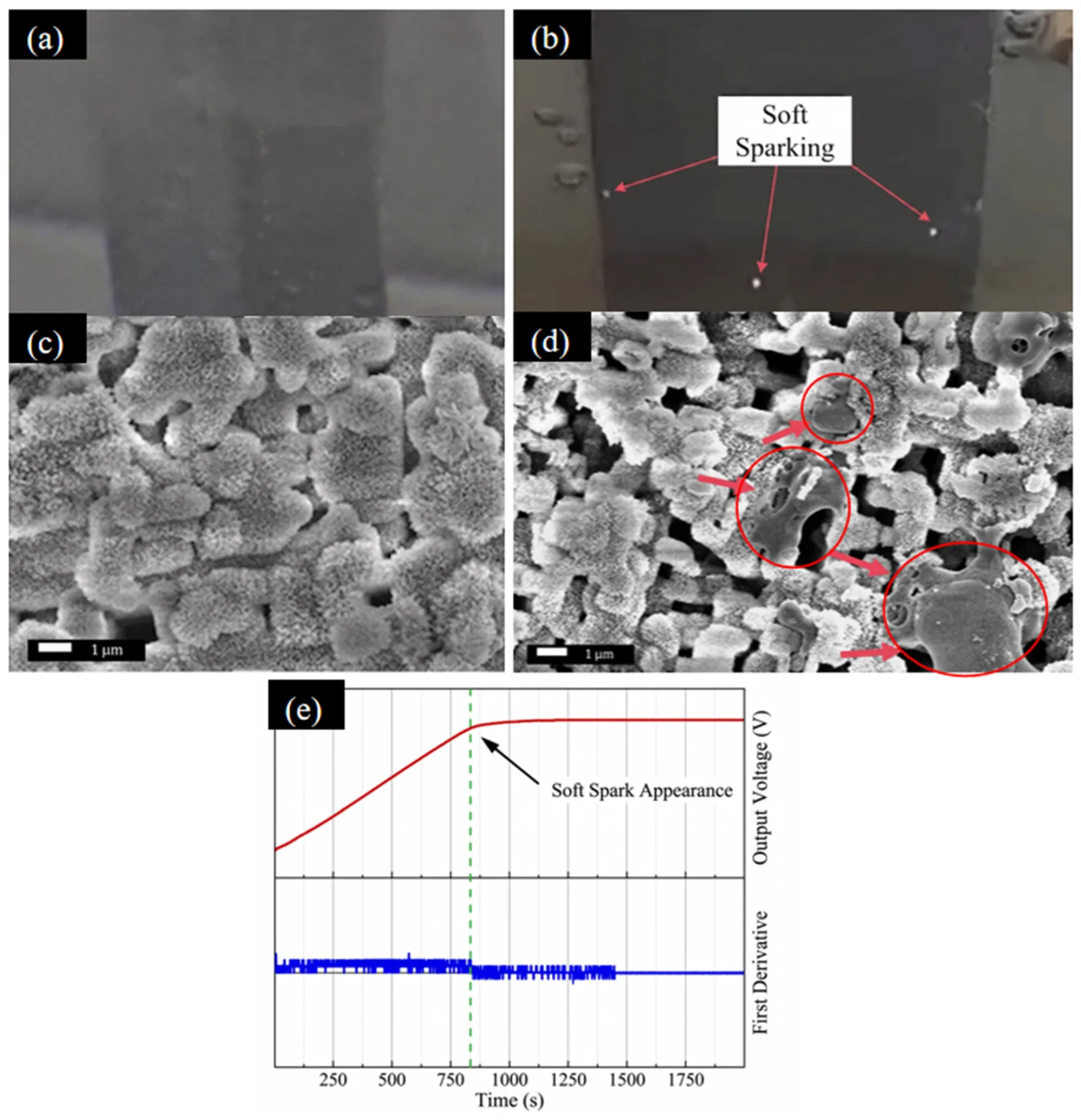
Surface morphology and microstructure comparison between conventional anodizing and soft sparking regimes: (**a**,**c**) no soft sparking; (**b**,**d**) with soft sparking. (**e**) Formation curves and their first derivative curves under high-conductivity voltage.

With increasing concentration of the boric acid electrolyte, the growth of the oxide film is promoted under an appropriate applied voltage, resulting in a dense and well-integrated film. However, improper matching between the applied voltage and the solution concentration can trigger soft sparking events, which may compromise the integrity of the oxide film. This leads to a repetitive sequence of dielectric breakdown and subsequent repair—i.e., breakdown, repair, re-breakdown, and re-repair—during film growth, thereby substantially increasing the overall energy consumption of the formation process. The soft sparking phenomenon is illustrated in Figure 3a–d (The soft sparking phenomenon is illustrated in Figure 3b,d, where the arrows indicate the sites at which soft sparking occurs, and the circled regions show the corresponding post-sparking characteristics of the oxide film).

At elevated electrolyte conductivities, spark discharge phenomena occur when the applied voltage reaches a certain threshold. These discharge events can be identified by processing the voltage data acquired during the formation process, as illustrated in Figure 3e. Specifically, by taking the first derivative of the formation voltage curve and analyzing the resulting derivative profiles (shown in Figure 3e), a clear decline in the voltage rise rate can be observed during the high-voltage stage following the onset of spark discharge, as indicated by the arrow in Figure 3e.

The phenomenon of soft sparking observed on the surface during the formation process is elucidated through the application of the Ikonopisov model [27]. According to the model proposed by Ikonopisov, the application of a sufficiently high voltage induces an electron avalanche at the interface between the formation solution and the oxide film [28], leading to dielectric breakdown. Furthermore, the breakdown voltage is inversely related to the logarithm of the conductivity of the formation solution, as illustrated in Equation (1):

(1) $$V_{1}=\alpha +\beta \log(\frac{1}{k})$$

In this context, *V*_1_ represents the breakdown voltage, while α and β are constant coefficients, and *k* denotes the conductivity of the formation solution. The application of this model demonstrates that an increase in the conductivity (*k*) of the formation solution results in a decrease in the breakdown voltage (*V*_1_). This paper reports the occurrence of dielectric breakdown at a formation solution conductivity of 2700 μS·cm^−1^, as evidenced by the appearance of spark discharge on the surface of the oxide film.

**Figure 4:**
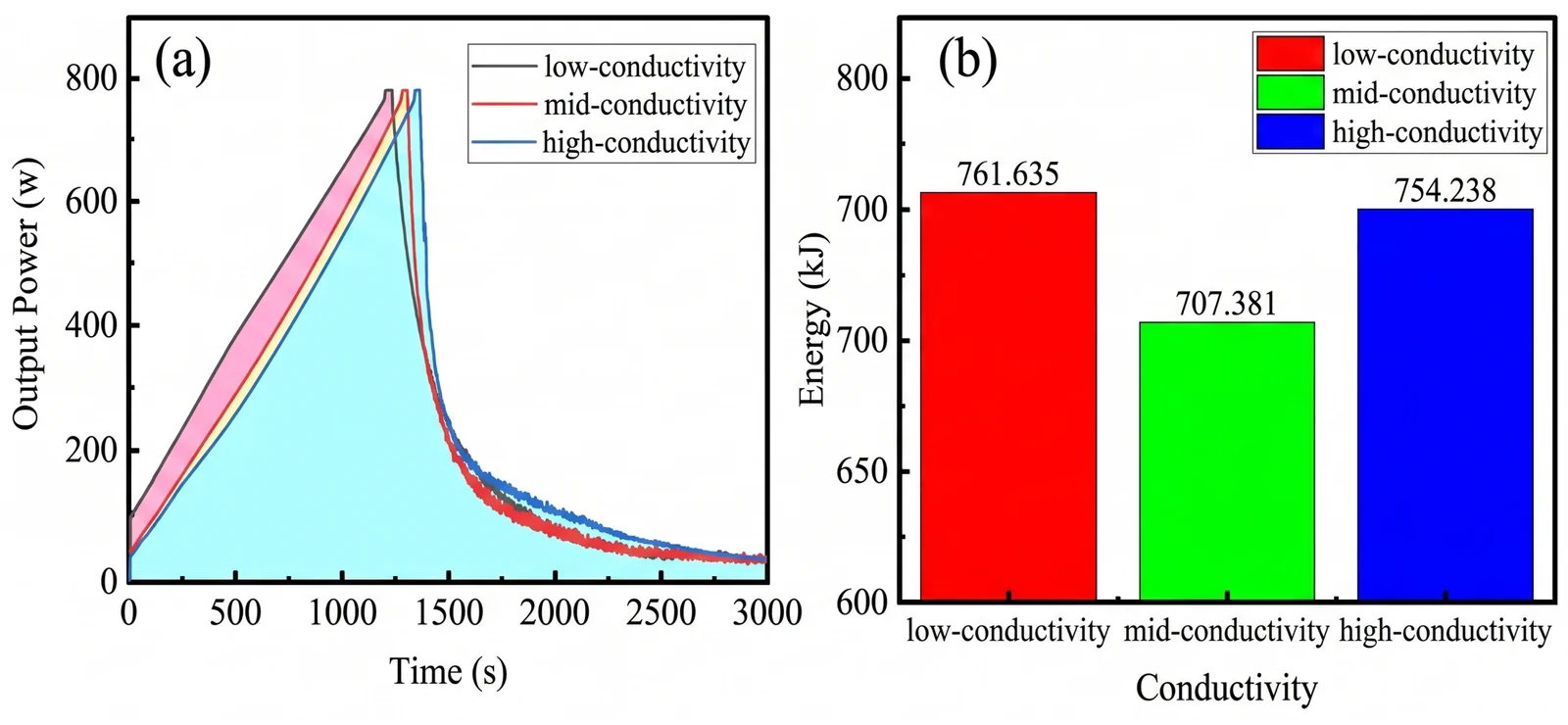
Formation power curves for the three conductivities (**a**) and energy consumption to form to 520 V (**b**).

To more definitively elucidate the influence of varying conductivities on energy expenditure during the formation process, an integration of the power curves was conducted. This analysis yielded energy consumption data for the formation process under three distinct conductivity conditions, as depicted in Figure 4. In systems characterized by high, medium, and low conductivities, the energy expenditure associated with the medium conductivity solution after 3000 s was measured at 707.381 kJ. This value represents a reduction of 7.1% and 6.2% in energy consumption compared to formations at low and high conductivities, respectively. The observed phenomenon can be attributed to the fact that an increase in conductivity facilitates the transfer of energy towards the enhancement of the oxide film’s growth, while concurrently reducing the energy expenditure related to the formation process. However, excessively high conductivity may lead to dielectric breakdown, resulting in soft spark phenomena during the formation process, which in turn increases energy consumption in high conductivity formations. Therefore, the energy consumption for formation at medium conductivity is significantly lower than that observed in both low and high conductivity formations.

**Figure 5:**
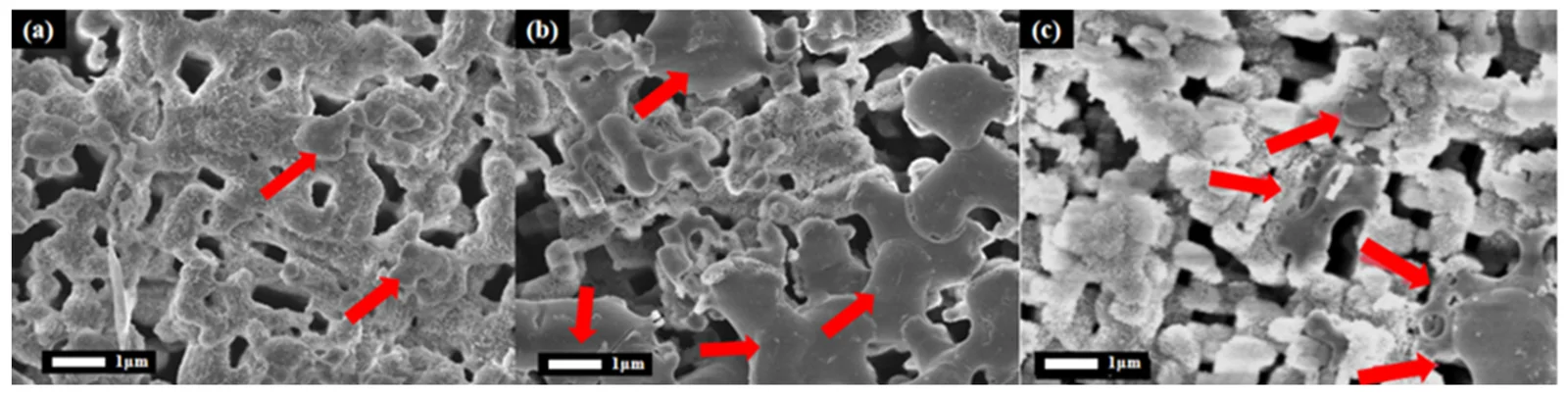
Surface microstructure of electrode foil after formation at 520 V under different conductivity levels (Arrows indicate the sites where soft sparking occurs): (**a**) 800 μS/cm; (**b**) 1500 μS/cm; (**c**) 2700 μS/cm.

The high energy consumption during conversion in high-conductivity conversion solutions stems primarily from two factors: First, during the discharge process, a portion of the energy is dissipated as Joule heating, resulting in energy loss. Second, the generation of spark discharges continuously damages the oxide film on the anode foil surface. As conductivity increases, the damage to the oxide film becomes more severe. The microstructural morphology of oxide films at different conductivity levels is shown in Figure 5. The arrow indicates the spark discharge zone, which necessitates increased energy input to repair the damaged oxide film.

In conclusion, within the context of low conductivity formation solutions, the combination of reduced ion concentration and elevated initial voltage facilitates the growth of the oxide film. This phenomenon results in increased energy consumption during the voltage rise phase of low conductivity formation when compared to medium and high conductivity formations. In the latter cases, a greater proportion of energy is expended to overcome the resistance of the oxide film, which is subsequently converted into thermal energy loss. While a higher concentration of boric acid can enhance the growth of the oxide film, excessively high concentrations can induce soft spark phenomena, thereby further elevating energy consumption during the formation process. Consequently, energy consumption remains relatively high in high conductivity solutions. Although medium conductivity formation exhibited the lowest energy consumption, the oxide film produced under these conditions demonstrated a lower degree of crystallinity.

### 3.2. Investigation of the Influence of Conductivity on Oxide Film Performance

**Figure 6:**
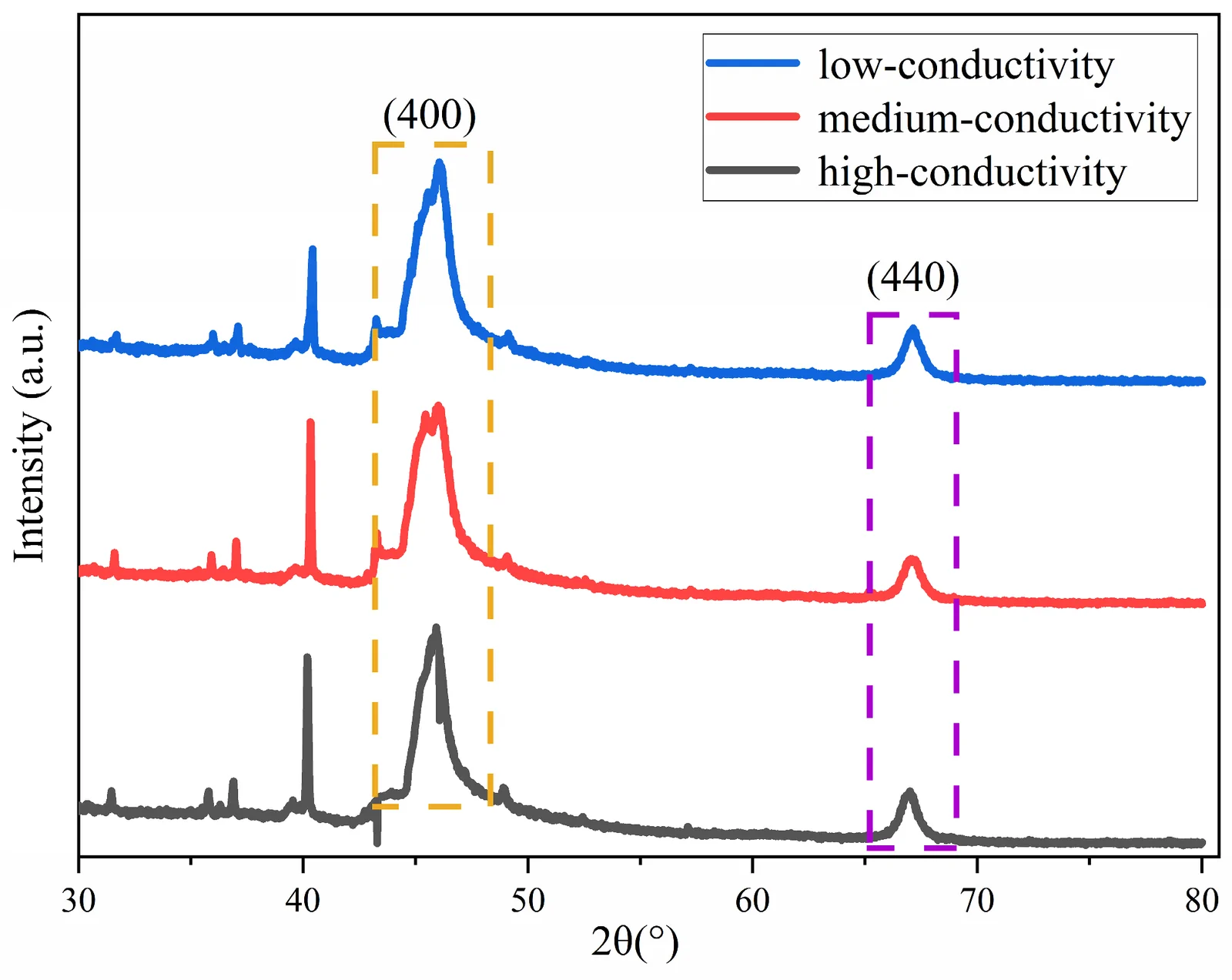
XRD patterns of the oxide films prepared at three different conductivities.

In practical industrial contexts, the energy expenditure associated with the formation process should not be regarded as the sole criterion for evaluation; the quality of the oxide film is also a crucial parameter that warrants substantial consideration. The oxide films produced under three different conductivity levels were analyzed using X-ray diffraction (XRD), with the results illustrated in Figure 6. A comparative analysis of the XRD data for the three oxide films indicated that a reduction in the peak at 66.893°—which corresponds to the (440) crystal plane of γ-Al_2_O_3_—was observed exclusively at the medium conductivity level. The peak intensity of the γ-Al_2_O_3_ (440) crystal plane is illustrated in the small box in Figure 6. The peak intensity at 66.893° for the high-conductivity sample is 875 counts, while it is 1014 counts for the low-conductivity sample and 836 counts for the medium-conductivity sample. This indicates that the medium-conductivity sample has the lowest crystallinity. The X-ray diffraction (XRD) data for the γ-Al_2_O_3_ (440) crystal plane are presented in Table 2. The full width at half maximum (FWHM) values for the high-, low-, and medium-conductivity samples are 0.84951°, 0.81578°, and 0.89305°, respectively. The crystallite sizes of the oxide films formed under the three conductivity conditions were derived from the full width at half maximum (FWHM) of the γ-Al_2_O_3_ (440) diffraction peak using the Debye–Scherrer equation. The FWHM values for the low-, medium-, and high-conductivity samples are 0.81578°, 0.89305°, and 0.84951°, respectively, yielding average crystallite sizes of 12.3 nm, 11.3 nm, and 11.9 nm, as summarized in Table 2. The underlying rationale for this observation is that, in a low conductivity electrolyte, the formation voltage rapidly reaches a steady state. Given that the overall duration of formation remains constant, the dwell time of the surface oxide film under stable current conditions is prolonged, thereby enhancing the crystallinity of the oxide film at the surface. In contrast, in a high conductivity formation solution, the phenomenon of dielectric breakdown results in sustained elevated current levels during the steady flow phase. During the dielectric breakdown process, a portion of the energy is utilized to induce the crystallization of the aluminum oxide film [29]. Consequently, higher crystallinity is observed in oxide films formed in high conductivity solutions. In contrast, in medium conductivity solutions, the increasing ion concentration facilitates the doping of ions into the oxide film more readily, which inhibits the film’s transition to a crystalline state, resulting in reduced crystallinity within the oxide film. This phenomenon leads to the lowest crystallinity in the oxide film at a conductivity of 1500 μS·cm^−1^ when compared to other conductivities of 800 μS·cm^−1^ and 2700 μS·cm^−1^. However, it is noteworthy that the energy consumption at a conductivity of 1500 μS·cm^−1^ is also the most reduced.

### 3.3. Optimization of Multi-Stage Formation Process

Previous studies have shown that high-conductivity electrolytes reduce Joule heating and improve energy efficiency during the initial stages of anodic oxidation by enhancing ion mobility [22]. Conversely, low-conductivity electrolytes have been demonstrated to promote denser oxide films with fewer defects during high-voltage formation, likely due to reduced ionic interference and controlled ion doping [30]. These findings directly support the design of the four-stage formation process, which combines high, medium, and low conductivity electrolytes to balance energy efficiency and oxide film quality.

Incorporating extensive research and analysis, while thoroughly evaluating energy consumption and the quality of the oxide film, the four-step formation conditions were designed based on the experimental results and literature review, which indicate that electrolyte conductivity significantly impacts both energy consumption and oxide film quality. In the low-voltage phase (up to 300 V), a high-conductivity electrolyte (2700 μS·cm^−1^) was employed to minimize thermal losses and accelerate oxide film growth. During the mid-voltage phase (300 V to 400 V), a medium-conductivity electrolyte (1500 μS·cm^−1^) was used to balance energy efficiency and oxide film crystallization. Finally, in the high-voltage phase (400 V to 520 V), a low-conductivity electrolyte (800 μS·cm^−1^) was applied to reduce ion doping and enhance oxide film crystallinity. This combination was selected based on the observed trade-off between energy consumption and oxide film performance in single-stage formation experiments. The specific parameters for the formation process are detailed in Table 3. Notably, since both the first and second formation stages fall within the low-voltage range (≤300 V), where film growth kinetics are primarily governed by the applied voltage, the same electrolyte conductivity (2700 μS·cm^−1^) is adopted for these two stages.

**Figure 7:**
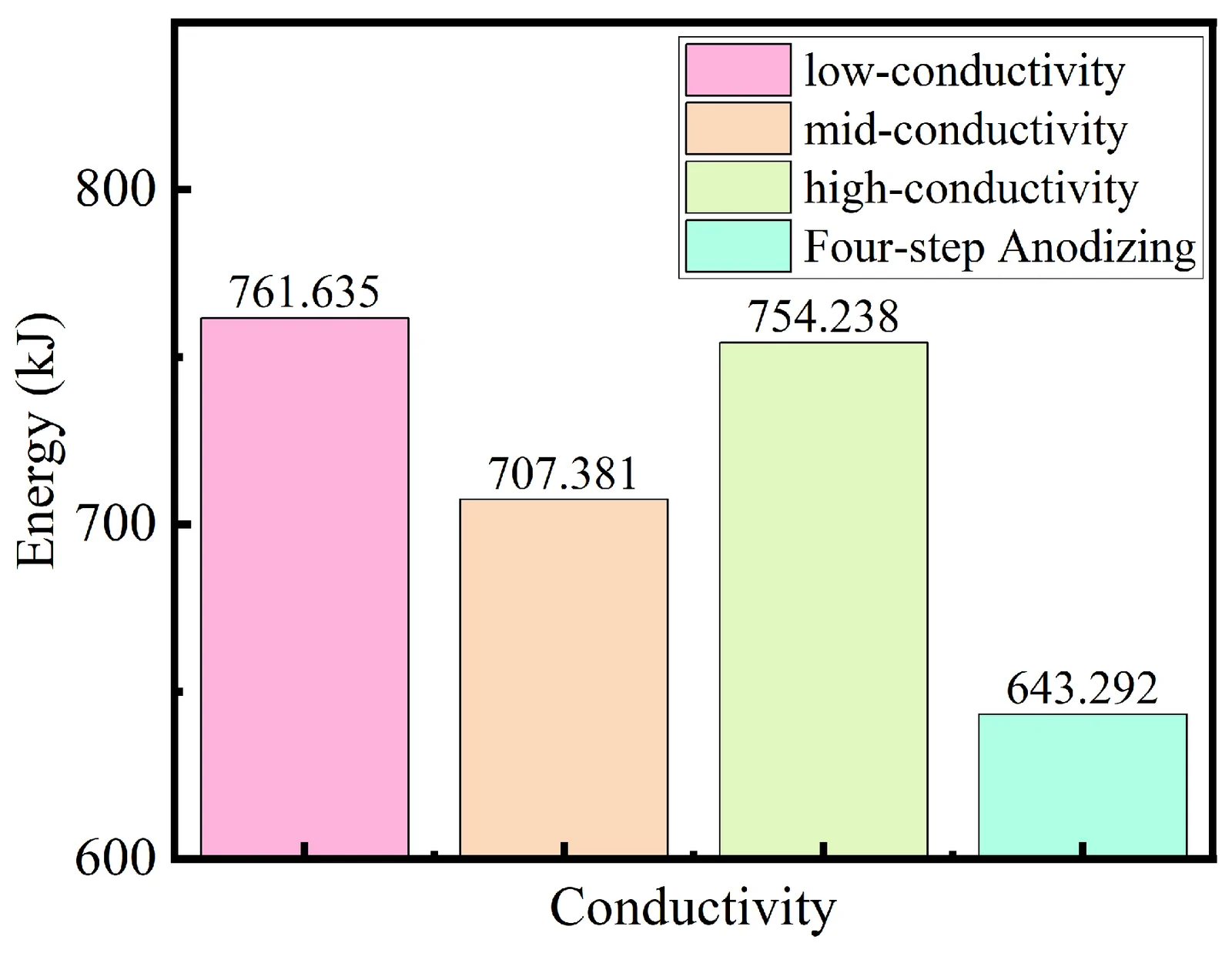
Bar chart comparing the energy consumption of the four-stage formation process with different conductivity single-stage formations.

As depicted in Figure 7, the energy consumption data pertaining to the quadruple-stage formation process is presented. The figure demonstrates that the utilization of a high-conductivity electrolyte during the low-voltage phase effectively minimizes thermal losses associated with oxide film resistance. Conversely, the application of a low-conductivity electrolyte in the high-voltage phase enhances the growth rate of the oxide film and mitigates the risk of dielectric breakdown. Notably, the four-step formation process results in an 8.4% reduction in energy consumption compared to the one-step process, signifying a substantial energy saving for the anode foil industry, which has an annual demand of 22.44 billion square meters.

An evaluation of the quality of the oxide film was conducted by analyzing the electrical performance data derived from one-step and four-step formation processes across various conductivities, with the results presented in Table 4. Tr (voltage rise time) represents the time required for the voltage to reach the target value, which is influenced by the growth rate of the oxide film. A shorter Tr indicates faster oxide film growth and fewer surface defects. Vt (voltage value) reflects the maximum voltage applied during the formation process, which affects the thickness and crystallinity of the oxide film.

As shown in Table 5, In comparison to the one-step formation process, the overall enhancement in specific capacitance and withstand voltage of the anode foil produced through the four-step formation is not substantial; however, the voltage rise time is notably shorter. The voltage ramp-up phase includes both the charging of the capacitor and the rectification of surface imperfections. Consequently, a more rapid voltage increase within a given timeframe suggests a higher quality oxide film with fewer defects. The ratio of Vt to Tr is employed to calculate the voltage escalation rate within a specified timeframe. In the one-step formation process, the anode foil prepared in a medium conductivity formation solution demonstrated the fastest ramp-up rate at 3.48 V/s. In contrast, the four-step formation yielded an anode foil with a ramp-up rate of 3.7 V/s, representing a 6% improvement over the rate achieved through the single-stage formation process.

## 4. Conclusions

This study clarifies the effects of electrolyte conductivity on energy consumption and oxide-film quality during the 520 V anodic formation of aluminum anode foil. During single-stage formation, low electrolyte conductivity (800 μS·cm^−1^) increased solution resistance and ohmic losses, whereas high conductivity (2700 μS·cm^−1^) promoted soft sparking and repeated breakdown–repair of the oxide film. Consequently, the medium-conductivity electrolyte (1500 μS·cm^−1^) achieved the lowest energy consumption of 707.381 kJ, which was 7.1% and 6.2% lower than those obtained at low and high conductivities, respectively. However, XRD results showed that the oxide film formed at medium conductivity had the lowest crystallinity, with a γ-Al_2_O_3_ (440) crystallite size of 11.3 nm. These results demonstrate that minimum energy consumption does not necessarily correspond to optimal oxide-film quality.

Based on this trade-off, a four-stage conductivity-switching process was developed by applying high conductivity at ≤300 V, medium conductivity at 300–400 V, and low conductivity at 400–520 V. This voltage-dependent strategy improves ion transport and reduces resistive losses during the low-voltage stages while suppressing ion incorporation and dielectric breakdown at high voltage. Compared with the conventional single-stage process, the optimized process reduced formation energy consumption by 8.4% and increased the voltage rise rate from 3.48 to 3.70 V·s^−1^, corresponding to an improvement of approximately 6%, while maintaining comparable capacitance and withstand voltage. Therefore, matching electrolyte conductivity with the applied voltage provides a practical approach to reducing formation energy consumption without compromising anode-foil performance.

## Figures and Tables

**Table 1:** Parameters for Single-stage Formation at Different Conductivities.

Formation Solution	Boric Acid Formation Solution
Conductivity of Formation Solution (μS·cm^−1^)	800/1500/2700 μS·cm^−1^
Thermal Processing Temperature (°C)	500 °C
Thermal Processing Time (min)	2 min
Additional Formation Time (min)	10 min

**Table 2:** Solution resistance (Rs) determined by EIS at different electrolyte conductivities.

Conductivity (μS·cm^−1^)	Rs (Ω)	Calculated iR Drop (V)	Initial Formation Voltage (V)
800	8.6	12.9	69
1500	4.2	6.3	34
2700	2.4	3.6	26

**Table 3:** XRD Data of the γ-Al_2_O_3_ (440) Crystal Plane.

Conductivity (μS·cm^−1^)	2θ	Intensity (Counts)	FWHM (°)	Crystallite Size (nm)
800	66.893°	1014	0.81578	12.3
1500		836	0.89305	11.3
2700		875	0.84951	11.9

**Table 4:** Multi-Stage Formation Conditions with Voltage-Dependent Conductivity.

	First-Stage Formation	Second-Stage Formation	Third-Stage Formation	Fourth-Stage Formation
Conductivity (μS·cm^−1^)	2700	2700	1500	800
Formation voltage (V)	150	300	400	520
Time (min)	15	15	10	10

**Table 5:** Electrical performance of anode foil from different conductivity single-stage formations and four-stage formations.

	Tr	Vt	Cap (μF)
low conductivity	162	540	4.13
medium conductivity	155	540	4.10
high conductivity	166	545	4.18
four-stage formation	147	544	4.11

## Data Availability

The original contributions presented in this study are included in the article. Further inquiries can be directed to the corresponding authors.
